# Performance Evaluation of Hot Mix Asphalt (HMA) Containing Polyethylene Terephthalate (PET) Using Wet and Dry Mixing Techniques

**DOI:** 10.3390/polym15051211

**Published:** 2023-02-27

**Authors:** Nisma Agha, Arshad Hussain, Agha Shah Ali, Yanjun Qiu

**Affiliations:** 1 School of Civil and Environmental Engineering (SCEE), National University of Sciences and Technology (NUST), Islamabad 44000, Pakistan; 2 School of Civil Engineering, Southwest Jiaotong University, Chengdu 610031, China

**Keywords:** eco-friendly, recycle, plastic bottles, polyethylene terephthalate (PET), hot mix asphalt (HMA), polymer-modified bitumen (PMB), fatigue life, moisture susceptibility, stability, flow

## Abstract

This study evaluates the performance of Polyethylene Terephthalate (PET)-modified hot mix asphalt. Aggregate, bitumen of grade 60/70 and crushed plastic bottle waste were utilized in this study. Polymer Modified Bitumen (PMB) was prepared using a high shear laboratory type mixer rotating at a speed of 1100 rpm with varying PET content of 2%, 4%, 6%, 8% and 10%, respectively. Overall, the results of preliminary tests suggested that bitumen hardened with the addition of PET. Following optimum bitumen content determination, various modified and controlled HMA samples were prepared as per wet and dry mixing techniques. This research presents an innovative technique to compare the performance of HMA prepared via dry and wet mixing techniques. Performance evaluation tests, which include the Moisture Susceptibility Test (ALDOT-361-88), Indirect Tensile Fatigue Test (ITFT-EN12697-24) and Marshall Stability and Flow Tests (AASHTO T245-90), were conducted on controlled and modified HMA samples. The dry mixing technique yielded better results in terms of resistance against fatigue cracking, stability and flow; however, the wet mixing technique yielded better results in terms of resistance against moisture damage. The addition of PET at more than 4% resulted in a decreased trend for fatigue, stability and flow due to the stiffer nature of PET. However, for the moisture susceptibility test optimum PET content was noted to be 6%. Polyethylene Terephthalate-modified HMA is found to be the economical solution for high volume road construction and maintenance, besides having other significant advantages such as increased sustainability and waste reduction.

## 1. Introduction

Plastic is a material that is consumed at an increasing rate every year. Its use is so common because of its good electrical and mechanical insulating properties, good chemical resistance, low density and easy processing along with its main advantage of less initial cost but the main problem lies in the disposal of this commonly used material. The improper disposal of plastic thus results in plastic pollution. Due to the non-biodegradable nature of plastic, it poses a serious threat to land and various waterbodies, because of which a considerable percentage of marine and land creatures are exposed to life-threatening situations [1]. Of all the various plastic types, plastic bottles exist untreated in large quantities at garbage dump sites, which threatens environmental safety. Plastic bottles, due to their chief chemical component Polyethylene Terephthalate (PET), are often regarded as PET bottles. The non-biodegradable nature of plastic bottles makes them lethal to animals when ingested. This is because the intermolecular bonding structure of PET is of the kind that does not allow it to decompose, digest or corrode. If improperly disposed of, these plastic materials pollute water bodies and become a cause of water-borne diseases. Consequently, water bodies are hindered by plastics in terms of their flow, which creates problems of pollution and suspension. These adverse environmental impacts of plastics necessitate their recycling or their use for some other purpose that can be beneficial as well as environmentally friendly. 

Besides environmental concerns, economic development and sustainability are of crucial importance to countries’ growth and overall national security. An important factor in improving a country’s economic status is that of the road infrastructure system and more importantly the length of existing paved roads, often used as an index to assess the extent of a country’s development. The presence of a proper road transport network not only minimizes the transportation cost, both in terms of time and financial aspects, but also aids in the interlinking of several regions within the country and in a better understanding of neighboring countries at a global level [2]. Transportation links (roads) are the prerequisite of a transport system. They carry traffic and continuously face load repetitions and, moreover, the damaging effect of climate results in various road defects.

One of the major deteriorations caused by the environment is the exposure of pavements to water, hence causing moisture damage. Moisture damage is defined as the loss in strength and durability of asphalt mixtures caused by the presence of water. The damage gains momentum as more moisture permeates and gradually causes the mastic to weaken, making it even more susceptible to damage during cyclic loading [3]. Poor drainage conditions or excessive rain can be the reasons for this uncontrolled exposure. Thus, pavements become more prone to cracking and other pavement distresses such as stripping, rutting, bleeding, corrugation and shoving, cracking, raveling and other localized failures [3].

**Figure 1:**
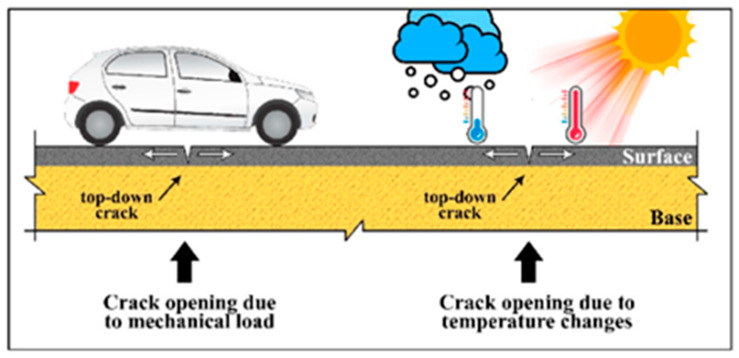
Pavement cracking.

Fatigue cracking in the bound layer is a major indication of structural failure in a pavement. The magnitude, frequency and duration of load application were identified to have major effect on pavement performance in terms of surface cracking [4]. Repeated traffic loads on pavements cause fatigue failure and because of that a series of interconnected cracks are formed on the road surface termed as fatigue cracking. For thin pavements, the initiation of cracking is from the bottom of the pavement as the tensile stress is greatest at that portion of the pavement. This cracking then propagates to the surface, which is commonly known as bottom-up fatigue cracking. For thick pavements, the cracking initiates from the top of the surface and from the surface areas of high localized tensile stresses, which exist because of the pavement–tire interaction. Moreover, the aging of the asphalt binder also plays an important role in top-down fatigue cracking in the case of thick Hot Mix Asphalt (HMA) pavements [5], see Figure 1.

Fatigue cracking reduces the overall life of pavements and results in major ride discomfort. These surface distresses also result in an increase in maintenance costs. Therefore, remedial measures that can address the issue of surface distresses because of increased traffic, vehicular loading and environmental deterioration can save the economy in terms of low initial project and rehabilitation costs. The utilization of plastics in road construction can serve the purpose to a substantial extent. The use of plastics in pavements has exhibited some very desirable results as far as pavement response to load repetitions and resistance to moisture damage is concerned. With the use of plastics in roads, the durability of the pavement structure can be increased, and waste plastic material can also be utilized that reduces the environmental threat. It is, therefore, the most suitable option to utilize waste plastic material in a way that contributes significantly in the field of road construction and plays a significant part in reducing environmental pollution.

Besides the eco-friendly disposal of plastic waste by incorporating it in road construction to mitigate the early deterioration of roads, the use of recycled plastic waste is also a cost-effective approach [6]. Waste polymers can be incorporated in HMA pavements through three techniques, wet mixing, dry mixing and aggregate replacement. In the wet mixing technique, PET is thoroughly mixed with hot liquid asphalt binder to form a mixture prior its mixing with aggregate. In the dry mixing process, to uniformly disperse the polymers, substantial mixing and shearing are required. For this method, polymers as solid granular particles or in the form of chips are mixed with the aggregates followed by the addition of bitumen [7]. As per Mishra and Gupta, the dry process is simple, economical and environmentally friendly, while the wet process requires more effort in terms of investment and machinery, and hence is not commonly used [8]. However, in this research for both mentioned techniques PET is used as a substitute material for a portion of the bitumen in Job Mix Formula (JMF) calculations [9,10].

In all the mixes not containing any plastic content, fatigue life was found to be minimum. In contrast, as per Casey et al., the addition of a plastic particle ingredient drastically increases the fatigue life of asphalt mixes. It is illustrated by lower stress levels ranging from 250 kPa to 350 kPa that the fatigue life of mixes with 1% plastic is increased twofold. Since PET particles do not melt due to their higher melting point, this results in the PET particles existing as partially rigid materials in the mixture, thus improving the flexibility of the mixture [11]. With an improved mixture, the crack creation and propagation in asphalt mixtures is postponed, which eventually leads to an increased higher fatigue life. Baghaee et al., in his research conducted in 2013, evaluated the indirect tensile fatigue strength of PET-modified HMA mixtures according to EN 12697, and compared them to results of controlled mix. It was concluded that, due to the modified mixture’s improved flexibility, crack initiation and propagation was delayed, which eventually indicated the higher fatigue life of flexible pavements [12].

**Figure 2:**
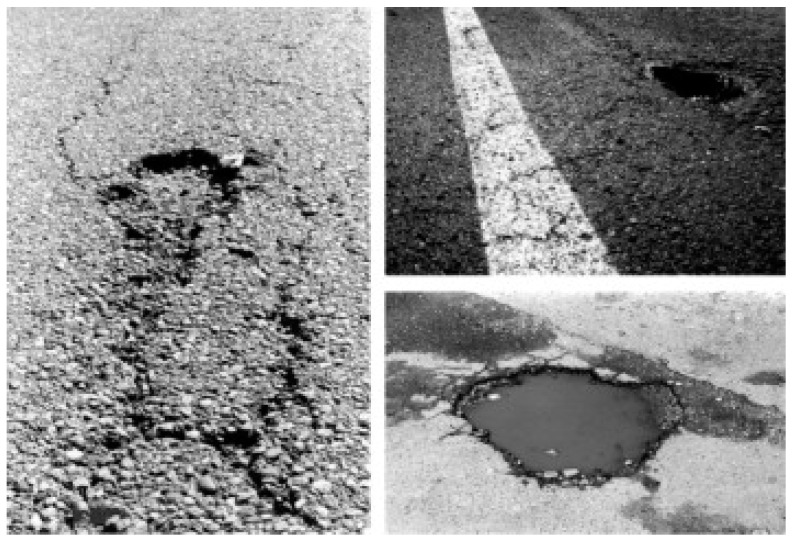
Moisture-induced pavement distresses.

As per Yilmaz et al., water damages the pavement in such a way that it decreases the strength and quality of asphalt concrete. Asphalt pavement is susceptible to moisture damage by the loss of the bond between binder and fine and coarse aggregate. This loss in bond results in the propagation of cracks along the pavement length. Moisture damage further aggravates as water penetrates through cracks and debilitates the mastic, making it more vulnerable to moisture amid the cyclic loading of vehicles [3]. Ferreira et al. conducted research in Brazil in 2022 and concluded PET produced positive effects on the moisture resistance of HMA; this led to the greater tensile strength of conditioned samples resulting in a higher Tensile Strength Ratio (TSR). Ferreira et al. further suggested that an improvement in TSR is indicative of increased asphalt binder–aggregate adhesion under moist conditions [13], see Figure 2.

For regions associated with extensive rainfall pavement deterioration, Ferreira et al. also proposed that resistance to moisture damage can be enhanced by the replacement of natural sand with recycled micro polyethylene terephthalate, thus demonstrating the feasibility of the practical application of blending PET in concrete asphalt paving [13]. 

Silva et al. suggested improved resistance against moisture damage when micronized PET was incorporated as a binder modifier in varying contents of 0, 4, 5 and 6% (by bitumen weight) [14]. Ghabchi et al. concluded that the micro nature of PET improves the viscosity and binder–aggregate adhesion; this is reflected as an increase in resistance to rutting, cracking and damage induced by moisture [15]. Studies by Likitlersuang et al. also suggested that resistance against moisture damage is improved due to an increase in adhesion between the binder and aggregate in asphalt under moisture exposition [16].

Successful past studies by Bamigboye et al. and Tayyab et al. on HMA modification by crumb tires, glass waste, biofuel, slag, fiber, lime, fly ash and sasobit also suggest PET as a potential modifier [17,18].

Burak Sengoz and Giray Isikyakar in 2008 showed that, based on the type of polymer and its content, the morphology and properties of the modified bitumen and the mechanical properties of HMA modified by polymer change. Samples with low polymer content exhibited the continuous dispersion of polymer in bitumen; however, the continuous dispersion of polymer has not been observed in samples containing high polymer content. Improvement in the conventional properties, by polymer modification, includes penetration, susceptibility to temperature, softening point, etc [19]. Ali et al. stated when grinded plastic is used as a modifier in bitumen in a replacement ratio of increments of 0.5% up to 2%, the results of index properties such as the flash point, softening point, fire point and penetration varied as compared to those of virgin bitumen. Moreover, regardless of the replacement ratio, modified bitumen yielded satisfactory performance [20]. The penetration values of Polymer Modified Bitumen (PMB) decreased as the PET content increased in the test conducted by Sojobi et al., which is an indication of increased stiffness and softening point; also, the tests indicated that because of the more stable asphalt, ductility values also increased with increasing PET content [21].

The engineering properties of Stone Mastic Asphalt (SMA) mixture incorporating waste PET were studied by Ahmad et al. in 2017. The study evaluated the mechanical properties of the asphalt mixture blended with PET in proportions of 0%, 2%, 4%, 6%, 8% and 10%. Based on the research by Ahmad et al., bituminous mixtures incorporating PET showed improved properties such as increases in stiffness, stability and viscosity. In other words, pavement can better withstand fatigue damage, thermal cracking, rutting and stripping. Asphalt modification by polyethylene yielded better resistance against fatigue and deformation [22]. In a previous study by Al-Hadidy et al. in 2009, the effect of polyethylene on the low temperature performance and moisture sensitivity of SMA mixtures was studied. It was concluded that such modified mixtures produced satisfactory performance in areas with extreme variance of temperature and heavy rain zones [23]. Studies conducted by Nishanthini et al. in 2020 suggested that resistance to water damage and fatigue cracking decreased when HMA was modified by 5–10% PET (by weight of binder) and less than 18% (by weight of fine aggregate). Marshall stability also improved upon such HMA modification. [24]

Jegatheesa et al. in 2018 stated that, for both Polymer Coated Aggregate (PCA) and Polymer Modified Bitumen (PMB), Marshall stability values showed an increase with increasing PET content. However, Air Voids (AV), which showed an increase in PCA with the increasing PET content, were seen to decrease in the case of PMB [25]. In a related study, Baghaee et al. found increased Marshall stability values for the addition of PET up to 0.6% (aggregate replacement). Furthermore, results obtained by Baghaee et al. showed that by the addition of plastic into the mixture the internal friction was reduced, eventually resulting in higher flow [12]. A study by Ahmadinia et al. in 2011 showed that stiffer mixtures were obtained with the addition of PET greater than 6 percent, which resulted in increased Marshall stability and decreased flow values, although increasing PET content beyond 6% resulted in a decline in the stability of the asphaltic mix, which consequently resulted in increased flow [26].

Another study by Kalantar et al. in 2012 also suggests that with the addition of polymer the flow of the mixture increases [7]. Ahmadinia et al. in 2012 suggested that adding waste PET between 2–10% by weight of bitumen content in SMA mixtures results in the improved adhesion of PET granules between the asphalt HMA and PET-modified HMA prepared by wet and dry mixing techniques based on various performance characteristics, which include moisture susceptibility, indirect tensile fatigue strength, stability and flow. The use of plastic waste encourages reduced plastic waste and promotes reduced initial costs; therefore, cost analysis was also carried out. Table 1 summarizes all the past studies conducted specifically for the PET modification of bitumen using different modification strategies.

## 2. Materials and Methods

**Figure 3:**
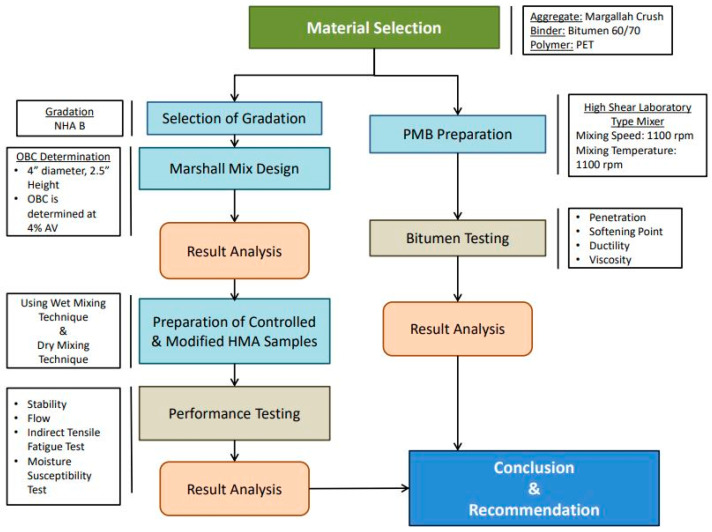
Research methodology flowchart.

Under the effect of vehicular loading and climatic changes, asphaltic pavements are prone to deteriorations. With increases in population and economic growth, demand for high quality roads has also increased. This demand is a big challenge to a country’s economy. This research aims at addressing the challenge faced by the construction industry and countries’ economies by suggesting a means to utilize plastic waste in road construction. The main objective of this research is to suggest the best mixing technique of PET in HMA mixtures by studying and comparing trends in the properties of virgin HMA mixtures with those of PET-modified HMA mixtures. The ITF strength and resistance to moisture damage are two key properties that are covered in this research besides assessing flow and stability. Figure 3 represents research methodology adopted for testing and analysis for this research. 

A testing matrix of the research was made for six different types of mixes that included one control mix and the remaining five were modified for varying PET content as shown in Table 2. Table 2 includes a series of various numbers of samples; the first mention of samples indicates the number of samples prepared for Type-x, the second mention indicates the number of samples prepared for Type-y and the third mention indicates the number of samples prepared for Type-z.

### 2.1. Binder

The base bitumen with a 60/70 penetration grade was selected in the current research due to adequate performance in cold to moderate climate conditions and was procured from Attock Refinery Limited (ARL), Rawalpindi, Pakistan. Conventional tests conforming to ASTM standards were performed to characterize the properties of base bitumen. The results obtained have been tabulated in Table 3.

### 2.2. Aggregate

Limestone aggregate procured from the Taxila (District Rawalpindi) quarry site was used in the current research. In order to find out the index properties of the aggregate, tests were conducted in conformity with the relevant test standards and are presented along with the results in Table 4.

**Figure 4:**
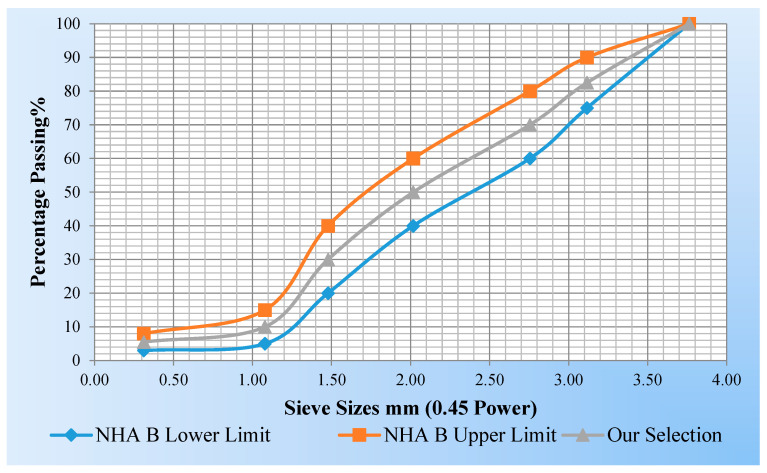
The NHA class-B gradation curve.

The gradation of the aggregate was chosen to conform with Pakistan National Highway Authority (NHA) standard Gradation-B for Asphaltic Concrete for Wearing Course (ACWC) as presented in Figure 4.

In addition to the gradation of aggregate, Table 5 shows blend ratios of 1200 g modified and controlled HMA samples. All quantities are measured in grams.

### 2.3. Polyethylene Terephthalate (PET)–Modifier

**Figure 5:**
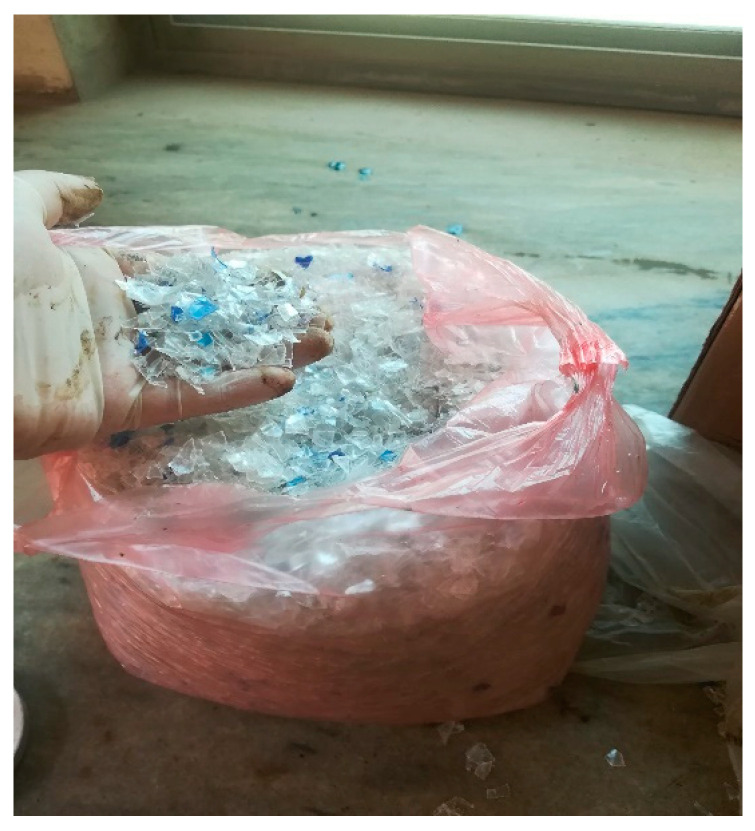
Crushed PET pellets.

Locally, waste plastic bottles, which were handpicked from streets, roads and public areas and sold by weight to local mechanical recyclers, were procured in the form of crushed pellets of size < 2.36 mm (see Figure 5). Typical properties of PET are tabulated in Table 6.

## 3. Sample Preparation

### 3.1. Preparation of PMB

Through the means of high shear laboratory type mixer revolving at around a speed of 1100 rpm, PET-modified bitumen samples were prepared. The base bitumen was brought to a fluid condition by heating it up to 155–160 °C. Later, PET was added gradually to the heated bitumen and mixing was continued for 2 h. Later, PMB samples of 2%, 4%, 6%, 8% and 10% PET content were stored in small containers and covered with aluminum foil for later testing.

### 3.2. Marshal Mix Design-OBC Determination

**Figure 6:**
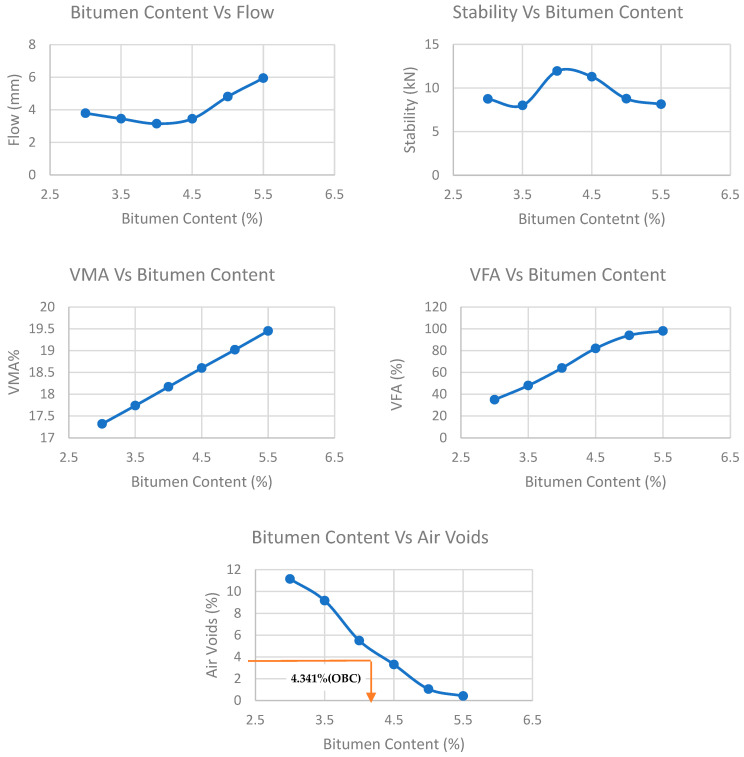
Volumetric properties of HMA samples.

The ASTM D-6927 standard for Marshall Mix Design was used to prepare controlled HMA samples. To determine the Optimum Bitumen Content (OBC), National Asphalt Pavement Association procedure was adopted. As per the procedure, HMA samples were prepared with varying bitumen content of 3.0%, 3.5%, 4.0%, 4.5%, 5.0% and 5.5%. A total of 1200 g of cylindrical HMA sample of 4” diameter and 2.5” height was prepared at a mixing temperature of 160 °C and compacted at 135 °C by giving 75 blows on each side of sample. Volumetric properties, which include Flow (mm), Stability (kN), Voids in Mineral Aggregate (VMA) (%), Voids Filled with Asphalt (VFA) (%) and Air Voids (AV) (%) of Marshall samples, were calculated and are plotted on graphs as shown in Figure 6. The HMA samples with higher bitumen content tend to have higher flow, VMA, VFA and lower stability and AV.

As per the National Asphalt Pavement Association, OBC was determined against 4% AV, which turned out to be 4.341%. All the other volumetric properties were then noted against 4% AV. Table 7 summarizes the volumetric properties of HMA at OBC.

#### 3.2.1. Controlled HMA Sample Preparation

As per ASTM D-6927, 1200 g of HMA samples were prepared by first heating aggregate and virgin bitumen up to 110 °C in an oven prior to mixing at 160 °C; the mix was later compacted to cylindrical samples at 135 °C by giving 75 blows on each side. Controlled HMA samples (Type-x) were prepared to assess properties at a later stage keeping in view Table 2.

#### 3.2.2. Modified HMA Sample Preparation–Wet Mixing Technique

A total of 1200 g of HMA samples were prepared by first heating aggregate and PMB (in% replacement of OBC) up to 110 °C in an oven prior to mixing at 160 °C; the mix was later compacted to cylindrical samples at 135 °C by giving 75 blows on each side. Modified HMA samples using the wet mixing technique (Type-y) were prepared keeping in view Table 2.

#### 3.2.3. Modified HMA Sample Preparation–Dry Mixing Technique

Keeping in view the high melting point of PET, the dry mixing technique was also considered. Aggregate was initially heated to a high temperature of 260 °C; later, crushed pellets of PET of a size < 2.36 mm were added and mixed thoroughly such that the aggregate was well coated with polymer; the mix was then cooled to 160 °C. Bitumen was separately heated up to 110 °C in an oven and then added to the heated aggregate-polymer mix at 160 °C. After the proper mixing of bitumen with aggregate-polymer, the mix was compacted to cylindrical samples at 135 °C by giving 75 blows on each side. Modified HMA samples using the dry mixing technique (Type-z) were prepared keeping in view Table 2.

## 4. Laboratory Testing–Results and Discussion

### 4.1. PMB Testing

**Figure 7:**
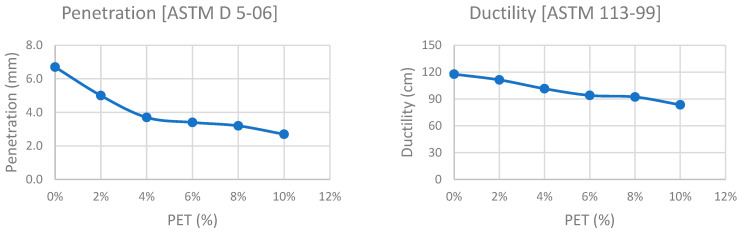

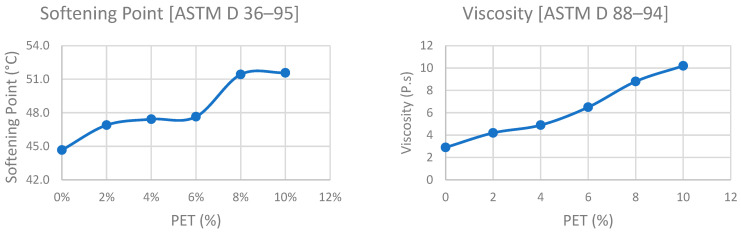
Preliminary Testing on PMB.

Various preliminary tests were conducted on the prepared modified bitumen samples as per the mentioned standards. The results of the virgin bitumen were also plotted for each index property to obtain a comparison between the controlled and modified samples of bitumen. Figure 7 represents trends in respective properties against each PET (%) content.

It is observed that an increase in PET content in PMB results in a gradual decline in ductility and penetration; however, softening point and viscosity increase. The increase in softening point suggests that bitumen resistance against the deformation of modified asphalt is improved. Preliminary testing conducted on PMB concluded that the properties of virgin bitumen altered noticeably when modified via PET.

### 4.2. Indirect Tensile Fatigue Test

**Figure 8:**
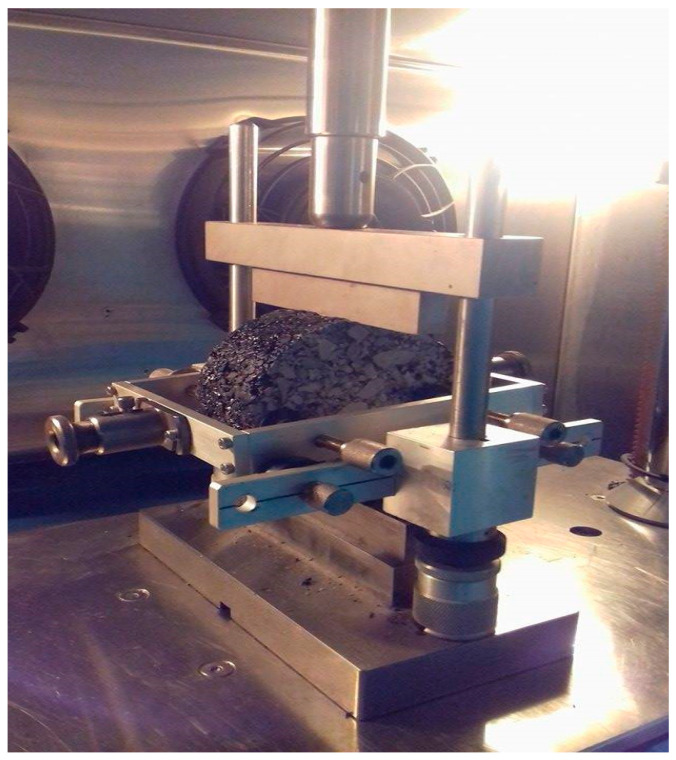
The ITFT load assembly.

**Figure 9:**
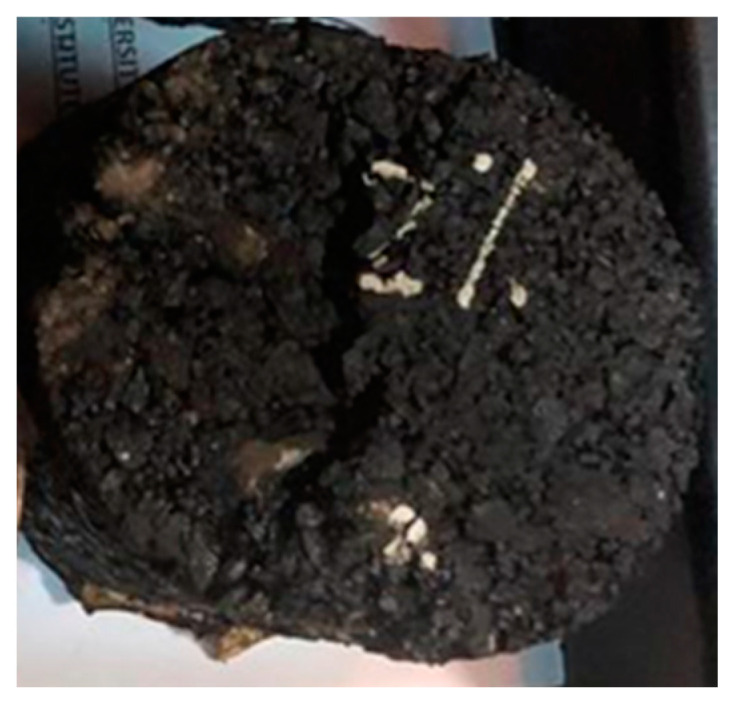
Fatigue-fractured sample.

The Indirect Tensile Fatigue Test (ITFT) was adopted to evaluate fatigue life in terms of cyclic loading in the Universal Testing Machine (UTM) following standard EN12697-24. The test was conducted in a stress-controlled condition such that the load was applied in a vertical direction resulting in horizontal tensile stress. The sample failed by splitting in the vertical plane due to increased strain within the sample [27]. Type-x, Type-y and Type-z of the HMA samples were tested for fatigue. As per EN12697-24, the thickness of the sample was 51 mm ± 1 mm and the diameter was 100 ± 3 mm (for a maximum aggregate size of 25 mm). Samples were conditioned for 4 h at 25 °C in a temperature-controlled chamber, prior to testing under a load of 3500 N, with a loading time of 0.1 s and rest time of 0.4 s. Figure 8 shows the device diagram and load symmetry of the ITFT. Testing was carried out at 25 °C. The test finished once the sample fractured, and the machine stopped itself. Figure 9 shows a fractured sample on completion of the loading cycles. For the sake of conclusion, the number of cycles leading to failure was noted. 

**Figure 10:**
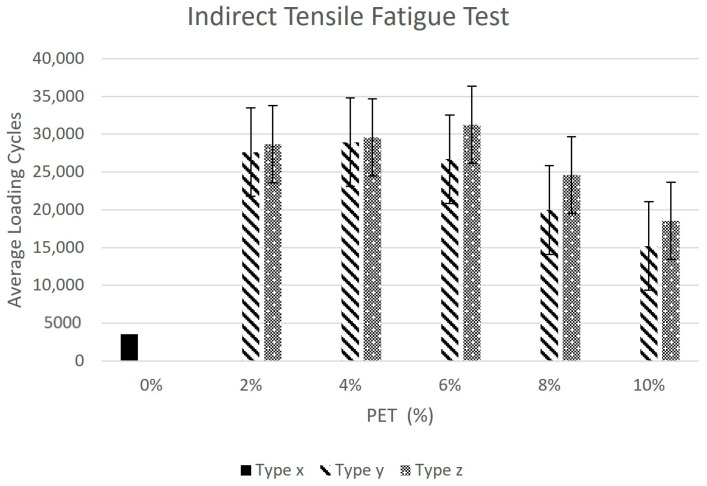
Indirect tensile fatigue test.

According to Baghaee et al., with the addition of plastic the fatigue life of asphalt mixes increases appreciably [12]. To get a traditional fatigue plot, loading cycles are plotted against varying PET content as shown Figure 10. This also represents a comparison of ITF loading cycles against each type of HMA sample (see Table 2) for varying PET content (%). The hypothesis is validated by the obtained results; fatigue life in terms of loading cycles increases dramatically with the increase in PET content from 0% to 2%; the increase is gradual as the PET content is further increased to 4% and 6% in the cases of wet mixing and dry mixing, respectively. However, fatigue life decreases as the PET content is further increased to 6% and 8% in Type-y and Type-z samples, respectively. It is to be noted that the dry mixing technique yielded better results in terms of fatigue life as compared to the wet mixing technique for each PET content.

As per regression analysis, the wet mixing technique produced a stronger relationship between average loading cycles, which signified fatigue life and variation in PET content. The R^2^ value for the wet mixing technique was 0.829 while for the dry mixing technique it was 0.613.

### 4.3. Moisture Susceptibility Test

In the past, various tests have been conducted to assess pavement susceptibility to moisture damage. As per Yilmaz et al., no test to date has accomplished any world-wide standardized acceptance to assess the extent of moisture-induced impairment. As a matter of fact, any test that can compare the test results of damp and dry HMA samples or establish a relation between the two can be utilized to assess the impact of moisture on HMA [3]. The Alabama Department of Transportation (ALDOT) provides a procedure to assess the resistance of HMA samples to moisture-induced damage as per standard ALDOT-361-88. This strategy assesses the change in diametral tensile strength caused by the saturation impact and conditioning of HMA samples on exposure to water [28].

**Figure 11:**
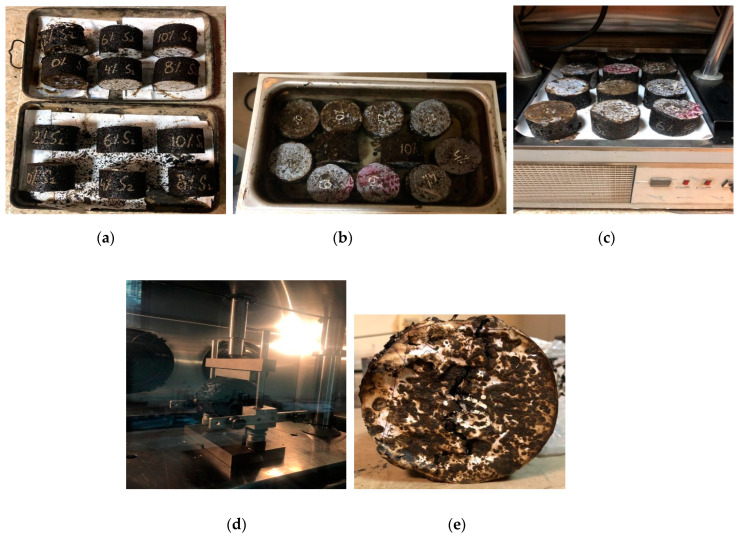
Moisture susceptibility testing: (**a**) Unconditioned samples ‘s-1’ and conditioned samples ‘s-2’; (**b**) Conditioning of samples in water bath for 24 h at 60 °C; (**c**) Unconditioned and conditioned samples kept at 25 °C for 4 h in a temperature-controlled chamber prior to testing; (**d**) Samples tested in UTM where the load was applied at 50 mm/min; (**e**) Fractured sample.

A sample diameter of 4” (100 mm) and thickness of 2.5” (63 mm) were used for moisture susceptibility testing in this research. For the controlled mix type (Type-x) two sets of samples were tested, conditioned and unconditioned samples. Similar types of samples were prepared to assess the moisture susceptibility of PET-modified HMA samples (Type-y and Type-z) with varying PET content of 2%, 4%, 6%, 8% and 10%, respectively (see Figure 11a). The samples were conditioned by placing them in a water bath at 60 °C for 24 h. (see Figure 11b). The samples were later placed at 25 °C for 4 h in a temperature-controlled chamber (see Figure 11c) and finally tested in UTM where the load was applied at 50 mm/min (see Figure 11d) Finally, maximum load leading to failure was noted in kN. Figure 11e shows fractured sample.

As per standard ALDOT-361-88, indirect tensile strength is measured for both conditioned and unconditioned specimens using Equation (1).

(1) $$S_{t}=\frac{2000*P}{\Pi *D*T}$$

Here:

$S_{t}$ = tensile strength (kPa);
*D* = sample diameter (mm);
*T* = sample thickness (mm);
*P* = maximum load (N).

The indirect tensile strength of conditioned and unconditioned samples was compared and presented in the form of TSR using Equation (2).

(2) $$TSR=\frac{S_{2}}{S_{1}}$$

Here:

$S_{2}$ = average tensile strength of conditioned sample;
$S_{1}$ = average tensile strength of un-conditioned sample.

**Figure 12:**
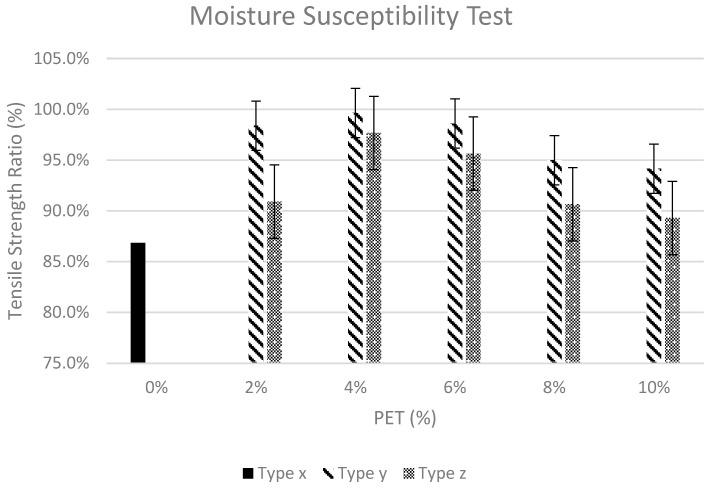
Moisture susceptibility test.

A minimum TSR value greater than 80%, adopted by many roadway agencies’ specifications [29], was observed for all three sample types. As per the research findings of Ferreira et al., Silva et al. and Ghabchi et al., the resistance against the moisture damage of PET-modified asphalt mixtures was noticeably higher as compared to controlled HMA mixtures [13,14,15]. Figure 12 shows a graphical representation of average tensile strength ratios for all three types of HMA samples containing varying PET content (%). The results as shown in Figure 12 also validate the hypothesis, which states that pavement tends to be less susceptible to moisture damage considering improved TSR (%), which increases considerably with the increase in PET content. The improvement in TSR (%) is gradual as PET content is increased from 0% to 2%. Furthermore, the increase in TSR (%) was noted to be gradual as the PET content was further increased to 4% in both mixing techniques. However, pavement resistance to moisture damage showed a declining trend as PET content was further increased beyond 4%. It is to be noted that the wet mixing technique yielded better results in terms of moisture susceptibility as compared to the dry mixing technique for each PET content. 

As per regression analysis, the wet mixing technique produced a stronger relationship between the tensile strength ratio and variation in PET content. The R^2^ value for the wet mixing technique was 0.734 while for the dry mixing technique it was 0.201.

### 4.4. Stability and Flow Test

**Figure 13:**
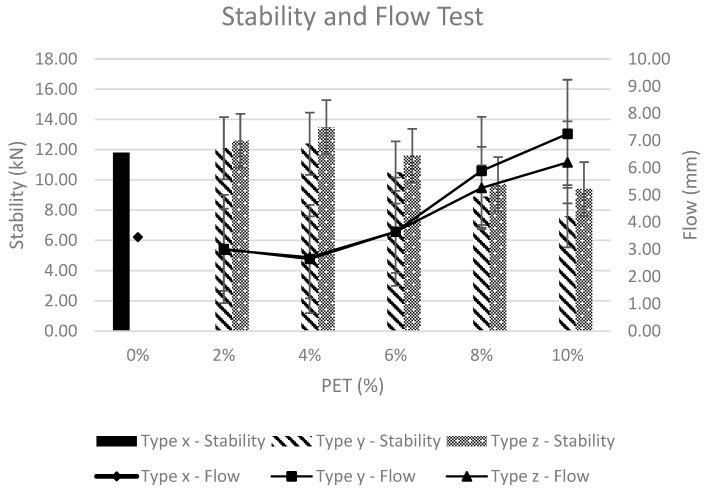
Stability and flow test.

As per AASHTO T245-90, the standard flow test and Marshall stability test were performed on a cylindrical sample. The specimen was placed in a water bath with a temperature of 60 °C for a period of 30 min, followed by samples being damp-dried and placed in the Marshall apparatus. Marshall stability is the maximum load applied for a given strain rate of 2 in. per minute, which brings about failure. During the stability test, the Marshall flow that occurs at a failure point is determined by a gauge that observes the vertical deformations, in mm, occurring in a specimen. The following Figure 13 shows a graphical representation of Stability (kN) and Flow (mm) with varying content of PET.

As per regression analysis, satisfactory results were obtained to establish the validity of both the wet and dry mixing techniques. The R^2^ value for the wet mixing technique in establishing the relationship between stability and variation in PET content was 0.922 while for the dry mixing technique it was 0.816; however, the R^2^ value for the dry mixing technique in establishing the relationship between flow and variation in PET content was 0.884 while for the wet mixing technique it was 0.865.

As per the research findings of Baghaee et al. and Ahmadinia et al., the stability of the asphalt mix increases with the addition of PET. The stability of PET-modified HMA is better as compared to controlled HMA samples due to improvement in adhesion among aggregate-binder-plastic pellets, [12,30]; however, in contrast to the findings of Ahmadinia et al. conducted in 2012, stability only increases to a maximum value corresponding to PET content of 4% [30] for both types of HMA mixes, i.e., Type-y and Type-z, and decreases gradually for higher PET contents. It is to be noted that Type-z HMA samples prepared by the dry mixing technique gave higher stability values at each PET concentration. At PET content ≥ 6%, the stability value falls to a value even less than Type-x stability.

High flow value is generally an indicator for a mix that is susceptible to permanent deformation under traffic; correspondingly, mixes with low flow values are indicative of the presence of higher air voids than normal values and pavement is likely to face premature cracking owing to the brittleness of the mix during the pavement’s service life. The research findings of Kalantar et al. conducted in 2012 and Baghaee et al. conducted in 2013 suggested that flow increases as PET content is increased, [7,12]; however, test results of Type-y and Type-z indicated a decrease in flow values up to the PET content of 4%, which later increased when PET content was increased beyond 4%. A similar trend was noted by Ali et al. in his research conducted in 2013 [20]; however, in the current research, the point of inflection for PET was noted to be 6%.

## 5. Environmental and Economic Sustainability

The usage of plastic in general and plastic bottles especially is on a surge throughout the world. Despite reducing-reusing-recycling efforts, plastic bottle waste is increasing day by day. As per the WWF study, total waste in Pakistan is estimated to be around 250 million tons of which a majority portion consists of food scraps, plastic bags and PET bottles. Plastic bottles produce 15.5% of total waste in Pakistan [31]. Pollution caused by PET is serious and growth in its consumption is alarming considering the long degradation period of around 300 years [32]. Furthermore, ever rising petroleum rates on a biannual basis [33], the depletion of natural resources [34] and the ban of limestone quarrying in the Margallah Hills [35], adverse environmental pollution, flooding, the lowering of water aquifers, high construction costs and lesser funds, the damage caused to pavements on exposure to moisture [3] and excessive and frequent vehicular loading [12] are a few of the many challenges faced by the road construction industry. Keeping his research findings in view, Baghaee et al. concluded that utilizing waste plastic as pavement-quality enhancer is rewarding, improving pavement life and serviceability while also preventing plastic waste from polluting the environment [12]. Ferreira et al. also concluded that the utilization of alternative pavement construction materials should be encouraged; this reduces the excessive utilization of raw materials and concurrently contributes to sustainable practices in engineering works [13].

**Figure 14:**
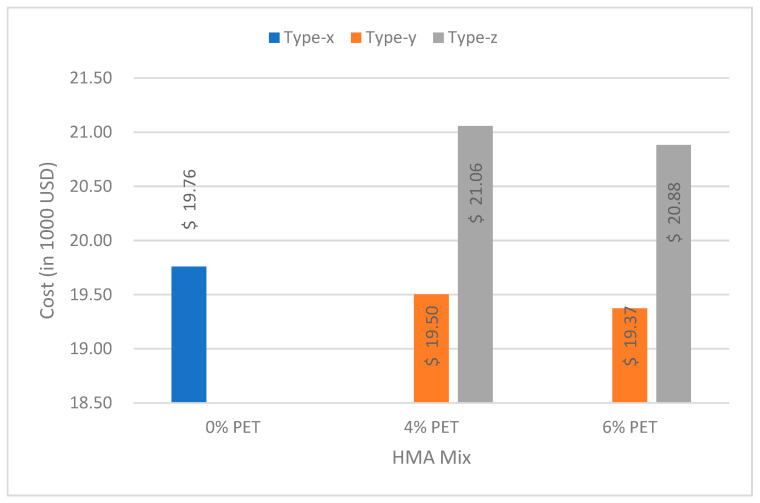
Cost comparison.

To assess cost reduction by using PET in HMA mixtures, cost comparison was carried out for virgin HMA and PET-modified HMA mix. A standard road width of 3.6 m for high-speed, high-volume highways [36] was assumed with an Asphaltic Concrete for Wearing Course (ACWC) thickness of 50 mm [37]. It was assumed for the lower layers of the two types of HMA mixtures, inclusive of Asphalt Concrete Base Course (ACBC), Granular Base Course (GBC), Granular Sub-Base Course (GSBC) that the preparation and compaction costs of the subgrade were completely similar and hence their cost was completely ignored. From the Marshall Mix design, the density of asphalt was found to be 2327 kg/m3 for the HMA mix. To estimate cost, the NHA’s section on the Composite Schedule of Rates (CSR)–2014 of the District of Rawalpindi was used [38]. Figure 14 indicates slight decreases of 1.30% and 1.95% in the cost for PET-modified HMA Type-y containing 4% and 6% PET, respectively, when compared with the job mix formula of virgin HMA for a one-kilometer road section. However, due to increased fuel consumption owing to high heating temperatures for the Type-z-modified HMA mix, the cost is 6.57% and 5.67% higher with 4% and 6% PET content. Kristjánsdóttir et al. in 2007 suggested the use of WMA technology, which can be incorporated successfully to lower the temperature at which asphalt mixtures are produced and paved [39]. It is to be noted that the cost analysis is only carried out for material costs involved at initial stages of road construction. When life-cycle cost assessment involving monetary and non-monetary costs is carried out, savings (%) will increase noticeably.

Table 8 illustrates various factors that have been considered for the cost analysis of the modified and unmodified mix.

## 6. Conclusions

This research evaluates the fatigue, moisture susceptibility, stability and flow properties of HMA modified by the addition of different percentages of PET to improve poor pavement serviceability due to high traffic volumes and weather conditions and to also assuage the growing pollution caused by plastic bottle waste. Therefore, the main objective of this research was developing environmentally friendly pavements by encouraging the reuse of waste material in the industry.

1. The PET modification of bitumen resulted in a higher softening point, penetration and viscosity of bitumen, which suggests that PET-modified pavement is less likely to be deformed. However, the ductility of PET-modified bitumen decreases gradually with increasing PET%.
2. With the addition and increase in PET content up to optimum level, the results for resistance against fatigue loading and moisture damage, as well as the results for stability and flow testing, improved. However, the performance testing results worsened once PET content increased further; this may be due to the decreased aggregate-binder bonding and increased stiffness of the modified HMA mix.
3. The dry mixing technique yielded better results for ITFT, stability and flow; however, the wet mixing technique produced better results for moisture susceptibility testing.
4. The optimum PET content that produced the best results for ITFT was 6% (dry mixing); however, for moisture susceptibility testing (wet mixing), flow and stability, the optimum PET turned out to be 4%.
5. Cost comparison suggests a 1.40% and 2.10% decrease in cost for the 4% and 6% PET-modified HMA mix prepared by the wet mixing technique, respectively. For the dry mixing technique cost increases by 5.55% and 4.60% for 4% and 6% PET content, respectively. The PET modification of pavement can be used to cut down life cycle costs. This will not only help to reduce road construction costs in the long run but can also help to mitigate environmental problems such as solid waste disposal and the depletion of natural resources.

This research can further be tested for dual wheel track laboratory equipment to assess the performance of PET-modified pavements under the rutting action of vehicles. The use of WMA technology might be helpful to reduce additional costs associated with the dry mixing of PET into HMA. Therefore, further testing is required to demonstrate the effectiveness of chemicals related to WMA technology and the interaction of such chemicals with the PET-HMA matrix. The current study can also be tested further by incorporating RAP in PET-modified HMA by following the homogenization treatment method for in-plant hot-mix recycled asphalt mixtures as conducted by Jie et al. in 2022 [40]. Furthermore, the current study could also be developed further by studying the change in properties of PET-modified HMA by partially replacing aggregate with slag as per the study conducted by Ahmad Goli in 2022 [41].

## Figures and Tables

**Table 1:** Past studies incorporating PET in asphalt mixes.

S. No.	Authors	Year/Country	PET Replacement Technique	Particle Nature	PET Modification Percentage	Conclusion
1.	Baghaee et al.	2013-Malaysia	Aggregate replacement	PET chips	0, 0.2, 0.4, 0.6, 0.8 and 1% (by aggregate weight)	Improved fatigue life of pavement upon addition of PET
2.	Ferreira et al.	2022-Brazil	Sand replacement	Crushed PET	2, 4, 8% (by sand weight) 8% (by sand volume)	Improved ITS and TSR of HMA improved when modified with PET; however, Resilient Modulus (RM) decreased
3.	Silva et al.	2018-Brazil	Binder additive	Micronized PET	0, 4, 5 and 6% (by bitumen weight)	Addition of PET improved results for Resilient Modulus, Indirect tensile strength, Lottman, Fatigue and Flow Number
4.	Ghabchi et al.	2021-United States	Binder additive	Micronized PET	0, 5, 10, 15 and 20% (by bitumen weight)	higher resistance to moisture- induced damage upon addition of PET
5.	Ali et al.	2014-Pakistan	Binder additive	Ground PET	0, 0.5, 1.0, 1.5, 2.0	Inclusion of PET improved test results for flash point, fire point, softening point and penetration test
6.	Sojobi et al.	2015-Nigeria	Binder modification Aggregate replacement	Molten plastic waste	0,5, 10, 20 (Polymer modified mix) 10, 20, 30 (polymer- coated)	Plastic content for PMB is likely to decrease penetration while increases softening point, ductility and viscosity. Stability and AV increased
7.	Ahmad et al.	2017-Malaysia	Binder additive		0, 2, 4, 6, 8 and 10% (by bitumen weight)	Improved stiffness, viscosity and rutting on addition of PET
8.	Jegatheesa et al.	2018-Sri Lanka	Binder additive	PET fibers with a nominal diameter of 0.5 mm and a length of 4.0 to 6.0 mm	5, 10, 15, 20, 25, 30, 35 and 40% (by bitumen weight)	Inclusion of PET improves Marshall stability and bulk properties
9.	Baghaee et al.	2013- Malaysia	Aggregate replacement	Crushed PET	0, 0.2, 0.4, 0.6, 0.8 and 1% (by aggregate weight)	On addition of PET, stability, flow and fracture resistance increased. Stiffness and specific gravity
10.	Ahmadinia et al.	2011-Malaysia	Binder additive	Sieve 1.18 mm passing; #40 retained	0, 2, 4, 6, 8, 10% (by bitumen weight)	Increased stiffness, Air voids and stability (up to 6% PET). Decreased bulk specific gravity
11.	Ahmadinia et al. in 2012	2012-Malaysia	Binder additive	Sieve 1.18 mm passing; #40 retained	0, 2, 4, 6, 8, 10% (by bitumen weight)	Addition of PET increased stiffness, resistance against rutting and provided lower binder drain-down

**Table 2:** Performance testing matrix.

S. No.	PET (% Bitumen)	Number of Samples Required		
		ITFT	Moisture Susceptibility	Stability and Flow
1	0	3+0+0	6+0+0	3+0+0
2	2	0+3+3	0+6+6	0+3+3
3	4	0+3+3	0+6+6	0+3+3
4	6	0+3+3	0+6+6	0+3+3
5	8	0+3+3	0+6+6	0+3+3
6	10	0+3+3	0+6+6	0+3+3
TOTAL		3+15+15	6+30+30	3+15+15

Type-x = controlled HMA samples. Type-y = modified HMA samples prepared using wet mixing technique. Type-z = modified samples prepared using dry mixing technique.

**Table 3:** Properties of base bitumen.

S. No.	Test Description	Specification	Results	Limits
1	Penetration test @ 25 °C	ASTM D 5-06	67	60–70
2	Flash Point (°C)	ASTM D 92	273 °C	~280 °C
3	Fire Point (°C)	ASTM D 92	375 °C	~320 °C
4	Softening Point (°C)	ASTM D 36–95	44.7 °C	35–45 °C
5	Ductility Test (cm)	ASTM 113-99	118 cm	>100 cm
6	Viscosity Test (Pa-sec)	ASTM D 88–94	2.98	≤3
7	Specific Gravity	ASTM D 70	1.02	0.97–1.02

**Table 4:** Properties of aggregate.

S. No.	Test Description	Specification		Results	Limits
1	Elongation Index (EI)	ASTM D 4791		11.20%	≤15%
2	Flakiness Index (FI)	ASTM D 4791		1.1%	≤15%
3	Aggregate Absorption	Fine:	ASTM C 128	1.6%	≤3%
		Coarse:	ASTM C 127	0.7%	≤3%
4	Impact Value	BS 812		15.23%	≤30%
5	Los Angles Abrasion	ASTM C 131		23.13%	≤45%
6	Specific Gravity	Fine:	ASTM C 128	2.12	-
		Coarse:	ASTM C 127	2.71	-

**Table 5:** The HMA blend ratios.

Controlled HMA Mix						
Aggregate	Bitumen Content					
	3.0%	3.5%	4.0%	4.5%	5.0%	5.5%
1/2	204	203	202	201	200	198
3/8	146	145	144	143	143	142
#4	233	232	230	229	228	227
#8	233	232	230	229	228	227
#16	233	232	230	229	228	227
#200	52	52	52	52	51	51
pan	64	64	63	63	63	62
Bitumen	36	42	48	54	60	66
**Modified HMA Mix**						
Aggregate	PET *w*/*w* of OBC					
	0%	2%	4%	6%	8%	10%
1/2	201	201	201	201	201	201
3/8	144	144	144	144	144	144
#4	230	230	230	230	230	230
#8	230	230	230	230	230	230
#16	230	230	230	230	230	230
#200	52	52	52	52	52	52
pan	63	63	63	63	63	63
Bitumen	51.6	50.6	49.5	48.5	47.5	46.4
PET		1.03	2.06	3.10	4.13	5.16

**Table 6:** Typical properties of PET.

S. No.	Property	Specification
1	Chemical Formula	(C_10_H_8_O_4_) n
2	Melting Point	260 °C
3	Typical Injection Mold Temperature	74–91 °C
4	Heat Deflection Temperature	70 °C at 0.46 MPa
5	Tensile Strength	152 MPa
6	Flexural Strength	221 MPa
7	Specific Gravity	1.56
8	Shrink Rate	0.1–0.3%

**Table 7:** Volumetric properties of HMA at OBC.

S. No.	Property	Value
1	Stability	11.7 kN
2	Flow	3.4 mm
3	VFA	70%
4	VMA	18.4%

**Table 8:** Cost analysis data.

Basic Parameters					
Segment length	1000 m				
Segment width	3.65 m				
Segment thickness	0.05 m				
Material volume	180 m^3^				
Material density	2327 kg/m^3^				
Material mass	418,860 kg~418.86 ton				
USD 1	PKR 226.52 (December 2022)				
Fuel price (Diesel)	PKR 235.30/liter (December 2022)				
Material	Type-x	Type-y		Type-z	
		4% PET	6% PET	4% PET	6% PET
Required binder (ton)	18.2	17.5	17.1	17.5	17.5
Cost of binder (PKR)	2,000,325	1,920,312	1,880,305	1,920,312	1,920,312
Required aggregate (ton)	400.7	400.7	400.7	400.7	400.7
Cost of aggregate (PKR)	1,541,081	1,541,081	1,541,081	1,541,081	1,541,081
Fuel required (liter)	3972.3	3972.3	3972.3	5561.2	5561.2
Cost of Fuel (PKR)	934,672	934,672	934,672	1,308,541	1,308,541
PET required	-	0.73	1.09	0.73	1.09
PET cost	-	21,814	32,721	21,814	32,721

## Data Availability

The data presented in this study is available on request from the corresponding author.
